# Profiling circulating microRNAs in maternal serum and plasma

**DOI:** 10.3892/mmr.2015.3879

**Published:** 2015-06-03

**Authors:** QINYU GE, YANTING SHEN, FEI TIAN, JIAFENG LU, YUNFEI BAI, ZUHONG LU

**Affiliations:** 1 Key Lab for Child Development and Learning Science, Ministry of Education, Research Center for Learning Science, Southeast University, Nanjing, Jiangsu 210096, P.R. China; 2 State Key Laboratory of Bioelectronics, Southeast University, Nanjing, Jiangsu 210096, P.R. China

**Keywords:** microRNA, serum, plasma, sequencing, pregnancy

## Abstract

Serum and plasma are two of the most commonly used materials in clinical diagnosis and investigations. Whether differential nucleic acids exist between the serum and plasma, and the way in which they may be selected in clinical diagnosis and applications remains to be elucidated. The present study sequenced microRNAs (miRNAs) in the serum and plasma of pregnant females using next generation sequencing technology. Several differentially expressed miRNAs were also verified by reverse transcription-quantitative polymerase chain reaction (RT-qPCR). In total, 329 miRNAs and 193 miRNAs were detected in maternal serum and plasma. Differential expression and different types of miRNAs were found in the serum and plasma, among them, 19 were upregulated and 6 were downregulated in serum when compared with plasma with a fold change >2.0 (P<0.001). The results demonstrated that a number of miRNAs were differentially expressed in the serum and plasma, and several of the miRNAs expressed in the serum were absent in the plasma. The results obtained using RT-qPCR in the selected miRNAs were similar to these results, and indicated that the differential expression of miRNAs in the serum and plasma provide a guide for further investigation and clinical use. The results of the analysis also suggested that differentially expressed DNA and RNA in the serum and plasma of pregnant females may be a result of the differential expression of miRNAs.

## Introduction

Serum and plasma are two of the most commonly used materials in clinical diagnosis. It is known that serum, following the exclusion of fibrinogen and other blood clotting factors, is essentially plasma. Fibrinogen is a type of protein responsible for the coagulation of blood, by converting itself into fibrin, whereas plasma is defined as the medium of blood, in which red blood cells, white blood cells and other components are suspended (1).

The identification of cell-free nucleic acids in plasma and serum (2–4) has led to the detection of fetal DNAs and fetal RNAs in maternal plasma (5–7). However, few reports have demonstrated the differences between the plasma and serum, and the majority have predominantly selected plasma and serum randomly in clinical and academic investigations. Lo *et al* revealed no significant differences in the results obtained from the serum or the plasma (8), although other previous studies have revealed that serum fetal DNA was higher compared with plasma fetal DNA (5,9,10). However, there remains no consensus regarding the method of selection of nucleic acids for specific investigations. Therefore, systematic investigations at the level of the genome are required to determine differences in the expression of certain nucleic acids between the serum and plasma.

MicroRNAs (miRNAs) are common, 19–22 nucleotide long, non-coding RNA molecules, which post-transcriptionally regulate gene expression by base-pairing with the 3′ untranslated region of complementary messenger RNA targets (11,12). miRNAs have been identified in animal cells and are considered to be important in numerous vital processes, including embryonic development, cellular differentiation, proliferation and apoptosis (13). It has also been demonstrated that miRNAs may function as tumor suppressors and oncogenes (14). Given the importance of miRNAs in post-transcriptional regulation, identification of these differentially expressed miRNAs may assist in elucidating the molecular differences between the serum and plasma of pregnant females.

miRNA in the peripheral blood of pregnant females, as one of the circulating nucleic acids, has been an area of focus due to its importance in gene regulation (15) and its potential use in non-invasive prenatal diagnosis. The development of the next generation sequencing technology has provided a promising tool for whole genomic assays of circulating miRNAs. Differentially expressed miRNAs have been detected using sequencing in maternal plasma at differential gestational ages and inabnormal pregnancy in several associated studies (8,16–19). In the present study, the miRNA in the serum and plasma of pregnant females were sequenced using Sequencing by Oligonucleotide Ligation and Detection (SOLiD) technology. Identification of the differentially expressed miRNAs between the maternal serum and plasma may provide an important foundation for the future application of serum and plasma in clinical investigations.

## Patients and methods

### Patients

Plasma samples were collected from 12 pregnant females in the second trimester (24–28 weeks of gestational age), serum samples were collected from 20 pregnant females in the same trimester and three females contributed serum and plasma samples. All pregnancies were normal, uncomplicated singleton pregnancies with no fetal malformation. Written informed consent was obtained from all the patients involved in the present study and the study was approved by the Ethics Committee of Zhongda Hospital, Southeast University (Nanjing, China).

### Sample processing and miRNA extraction

The plasma and serum were separated from the blood samples, according to our previous study (20). Briefly, for the plasma samples, 2 ml blood was collected from each female, placed in EDTA-containing tubes and centrifuged at 4°C and 1,600 × g for 10 min, and then at 4°C and 16,000 × g for a further 10 min, in order to free the plasma from any remaining blood cells. For the serum samples, the blood was allowed to clot by leaving it undisturbed at room temperature for 30 min following collection of the whole blood, following which the clot was removed by centrifugation at 4°C and 3,000 × g for 10 min. The serum and plasma were carefully transferred into fresh tubes and stored at −80°C. All the plasma and serum samples were pooled, respectively, prior to sequencing. The miRNA was extracted from 5 ml of the pooled plasma or serum using an mirVana™ miRNA Isolation kit (Ambion, Austin, TX, USA) with minor modifications (3 ml TRIzol LS reagent was added to the plasma or serum samples prior to purifying with the column). The circulating miRNA was eluted to a final volume of 20 *µ*l, and ~500 ng of miRNA was obtained from each group. The quantity and quality of the obtained miRNA was measured using a NanoDrop ND-1000 spectrophotometer (NanoDrop, Wilmington, DE, USA).

### Small RNA library construction and high-throughput sequencing

Small RNA libraries were constructed based on ligase-enhanced genome detection technology using a SOLiD small RNA expression kit (Life Technologies, Grand Island, NY, USA) with minor modifications, including using a different Ligase (T4 RNA ligase 1; NEB, Ipswich, MA, USA). L-adaptors and R-adaptors (Takara, Bio, Inc., Dalian, China) were ligated to the miRNA samples, which were subsequently purified by size-selection using 6% polyacrylamide gel electrophoresis following each ligation. A six nucleotide barcode (Takara Bio, Inc.) was introduced during RT-qPCR amplification. These two prepared libraries and other encoded samples were pooled at the same concentration (50 pg/*µ*l) prior to emulsion PCR (21). All the procedures for template bead preparation were performed, according to the standard SOLiD protocol, and the prepared slides were analyzed on a SOLiD system V3 (Life Technologies), according to the multiplexing instructions.

### Read filter and small RNA annotation

For the deep-sequencing reads produced by the SOLiD sequencer, the sequencing reads were decoded using barcodes, consequently allowing one mismatch in each six nucleotide coding region. Those reads, which matched a barcode uniquely were used for mapping. Mapping of the SOLiD reads was performed using a SOLiD system small RNA analysis pipeline tool (RNA2MAP, version 0.5.0; Life Technologies). Following filtering of the other human non-coding RNAs (rRNA, snoRNA, snRNA and tRNA), raw expression values (read counts) were obtained by totalling the number of reads that mapped uniquely to the, miRBase release 14.0 (http://www.mirbase.org/ftp.shtml) and Human Genome RefSeq Hg19 (ftp://ftp.sanger.ac.uk/pub/gencode/Gencode_human/) reference databases.

To quantify and compare the expression levels of miRNAs across the datasets or samples, the raw expression profiles were normalized using linear transformations of each dataset. Only those miRNAs with count values >10 were selected to produce the novel and reliable expression profiles for further analysis. The expression profiles were normalized against the total counts of 1,000,000, according to their percentage spaces. To compare the differential expression of miRNAs in the serum compared with the plasma, two indexes were analyzed, and the P-value and the fold-change of logarithmic analysis of the read counts (log2-ratio) were determined. Only those miRNAs with P<0.01 and fold-change >2 were considered to be differentially expressed in the sample compared with the control.

### Functional enrichment analysis of predicted target genes of differentially expressed miRNAs

Subsequently, the experimentally validated target sites were collected from the TargetScan database (22). The functional relationship of genes that are targeted by ≥2 miRNAs was evaluated using gene ontology (GO) and Kyoto Encyclopedia of Genes and Genomes (KEGG). To identify KEGG pathways enriched with gene targets, the gene set analysis was performed using WEB-based GEne SeT AnaLysis Toolkit (Web-Gestalt; Department of Biomedical Informatics, Vanderbilt University, Nashville, TN, USA). Statistical significance of pathways was determined on the basis of P<0.05 and the presence of at least two target genes in the pathway.

### Reverse transcription-quantitative (RT-q)PCR

The miRNA was extracted from the plasma and serum samples following pooling, according to the method described above. For the RT reactions, ~150 ng small RNA was used with an AMV Reverse Transcriptase XL kit (Takara Bio, Inc.) at 42°C for 60 min with a final volume of 20 *µ*l, followed by final incubation at 95°C for 5 min. The following qPCR was performed using SYBR^®^ Premix Ex Taq™ II (Perfect Real Time; Takara, Bio., Inc.) on an Applied Biosystems 7500 real-time PCR machine (Life Technologies) by using 2 *µ*l of the cDNA obtained in the RT reaction. The primers were synthesized by Takara Bio, Inc.. The PCR reaction was performed at 95°C for 5 min, followed by 45 cycles at 94°C for 15 sec, 58°C for 30 sec and 72°C for 30 sec. Each PCR was repeated three times. The relative expression level of each miRNA was normalized against that of U6 snRNA. The mean value of Ct for each triplicate was calculated. Fold changes in gene expression were calculated by ΔΔCt (23) and normalized with ΔCt = Ct_miRNA_ − Ct_U6_, ΔΔCt = ΔCt_serum_ − ΔCt_plasma_. Quantification of qPCR was based on determination of the quantification cycle and PCR efficiency was calculated from the log-linear portion of the standard curves. P-values of differentially expressed miRNAs were calculated based on Poisson's distribution. P<0.05 was considered to indicate a statistically significant difference.

## Results and Discussion

### SOLiD sequencing results of maternal serum and plasma

**Figure 1:**
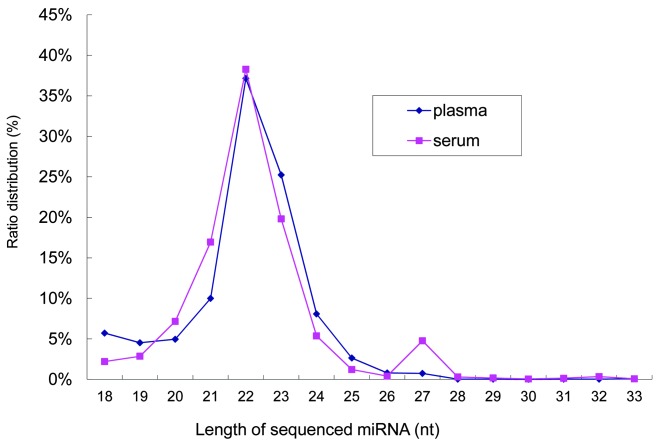
Length distribution of the sequenced miRNAs in the plasma and serum samples of pregnant females. miRNA, microRNA; nt, nucleotides.

To investigate small RNA transcriptions, SOLiD high-throughput sequencing was performed in serum and plasma small RNA libraries. The results demonstrated that ~47% of the decoded sequences were mappable using the SOLiD system small RNA analysis pipeline tool (RNA2MAP, version 0.5.0). Following filtering and removal of reads with low quality and no insert between the L-adaptor and R-adaptor, 2,925,149 and 6,850,295 sequence reads were obtained from the plasma and serum, respectively. On examining the length distribution of the small RNAs, it was revealed that the majority of the small RNAs from the two libraries were 22 nucleotides in length (Fig. 1), which was consistent with the typical size of miRNA from Dicer digestion products. These results indicated that the miRNAs had been successively enriched from the two libraries.

**Figure 2:**
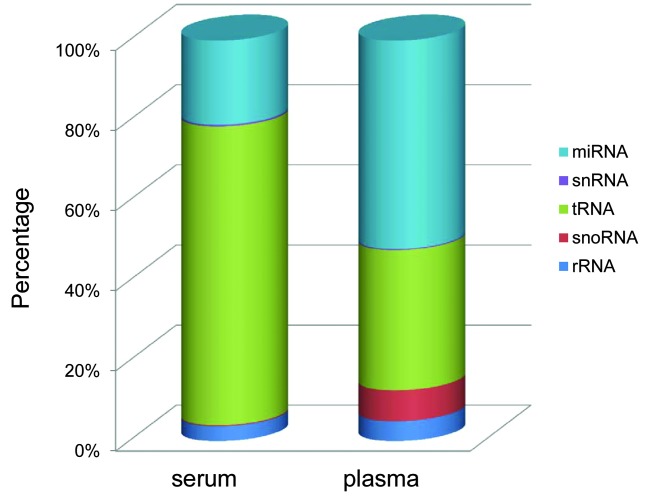
Distribution of differential small RNA species in the maternal serum and plasma according to the reads obtained by deep sequencing.

Based on human genome annotations and a number of well-characterized RNA databases, these small RNA sequences were annotated as known miRNA, tRNA, rRNA or snRNA/snoRNA. The most abundant RNA category from the plasma was a the known miRNA, which was 52.04%, as expected. However, the percentage of known miRNAs in the serum was only 21.02%, and the most abundant RNA category was tRNA, at 74.61% (Fig. 2). The other less abundant category, in the plasma library was tRNA.

### Differential expression of maternal serum and plasma miRNA

**Figure 3:**
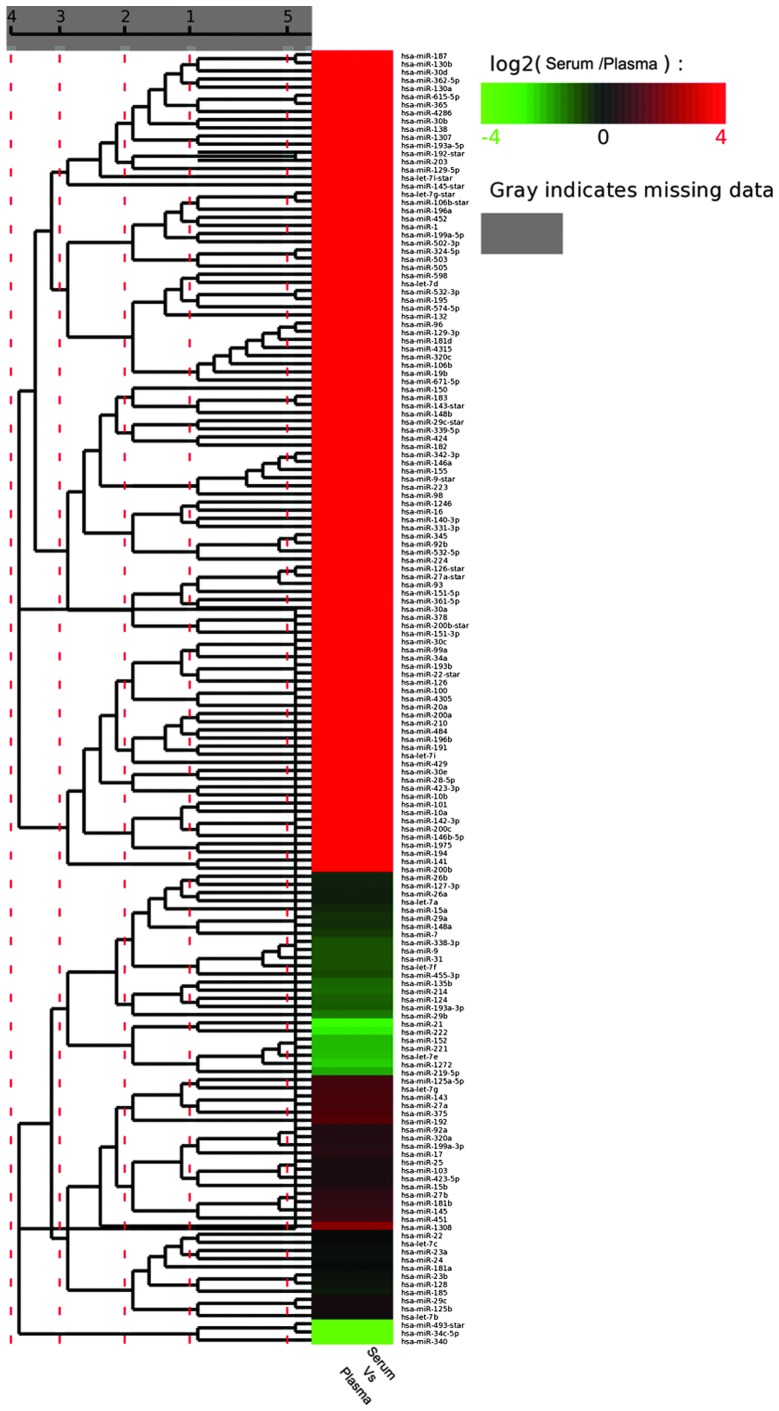
Hierarchical clustering analysis of differentially expressed miRNAs. Differential expression of miRNA in the serum, compared the plasma samples from pregnant females. miRNAs with a low copy number (count value <10) were removed from the data. The horizontal line above indicates the fold change of each miRNA. The color of each pattern represents the fold change as log2, with high indicated in red and low indicated in green. Gray indicates missing data. miRNA, microRNA.

Compared with the plasma samples, upregulation and down-regulation in the expression levels of miRNAs were observed in the serum samples of the pregnant females. Significant changes in the expression levels of the sequenced miRNA are shown in Fig. 3, determined using hierarchical clustering analysis. The color represents the differential expression of miRNAs between the serum and plasma samples, with red indicating upregulated expression and green indicating down-regulated expression of specific miRNAs.

A total of 329 miRNAs (276 miRNAs and 53 miRNAs^*^) and 193 miRNAs (171 miRNAs and 22 miRNAs^*^) were detected in the maternal serum and plasma, respectively. miRNA^*^ is a product produced from pre-miRNA. miRNA and miRNA^*^ are complementary in the stem-loop of pre-miRNA. The present study demonstrated that the differential miRNAs exhibited significantly differential expression levels, as measured by the frequency of read counts, indicating a marked functional divergence of these miRNAs. In the serum, ~9.12% of the miRNAs and miRNAs^*^ exhibited high read counts (>500), among which the hsa-let-7 family, comprising hsa-let-7c, hsa-let-7f, hsa-let-7a, hsa-let-7d, hsa-let-7b and hsa-let-7e, was one of the most abundant miRNAs in the data set.

**Figure 4:**
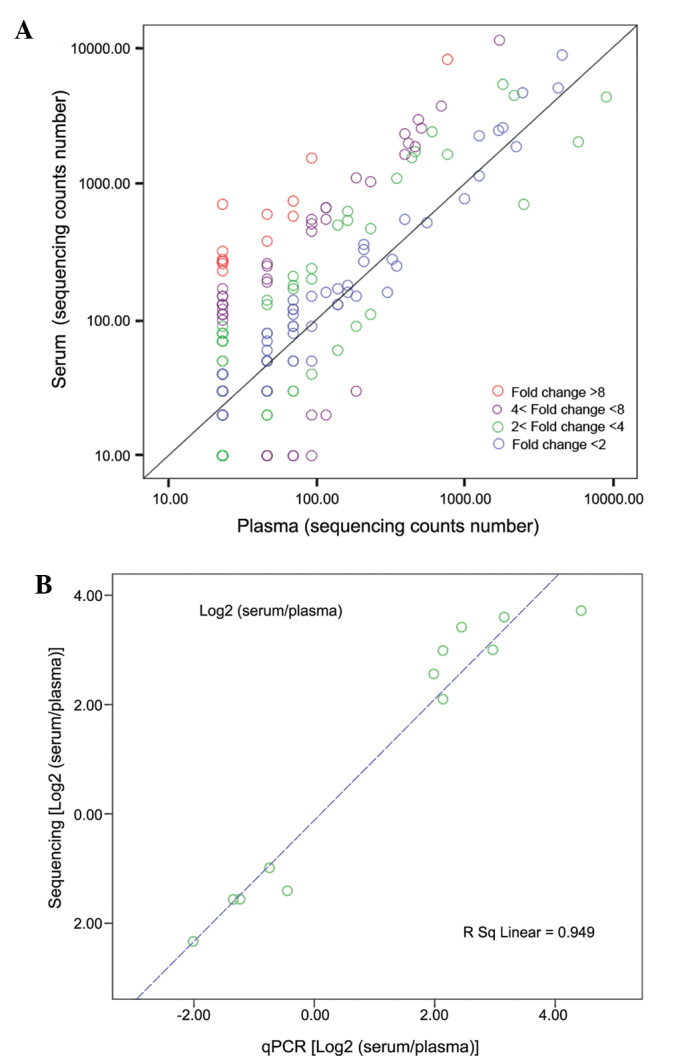
miRNA expression levels and validation of the robustness of SOLiD sequencing using RT-qPCR analysis. (A) miRNA expression levels in the maternal serum and plasma. The count of each miRNA was plotted following normalization. The colored circle indicate differentially expressed miRNAs between the maternal serum and plasma. (B) Comparison of SOLiD sequencing with RT-PCR of miRNA fold-changes. RT-qPCR, reverse transcription-quantitative polymerase chain reaction.

The relative count of sequencing reads was used to quantify the miRNA expression levels between the serum and plasma. Based on the normalized number of reads per sample (specific miRNA / total sequencing tags in the library), the miRNAs sequenced from the two libraries were counted. A total of 36 and 113 abundant miRNAs (original counts >20) were detected from the maternal plasma and serum, respectively. The miRNAs sequenced from the maternal plasma were all identified in the maternal serum, however, 77 miRNAs were only sequenced from the maternal serum. Among the 36 miRNAs, which were detected in the plasma and serum, 25 were identified to be differentially expressed, exhibiting fold changes >2.0 and P<0.001. (Table I; Fig. 4A), 19 were upregulated and six were downregulated. Among these, hsa-miR-1308 was identified as a fragment of tRNA and was excluded. The 20 miRNAs exhibiting the highest levels of expression in the two libraries are shown in Table I. The majority of the abundant miRNAs in the maternal plasma were also observed in the maternal serum.

### RT-qPCR verification

To further validate the differentially expressed miRNAs identified, 12 individual miRNAs were selected for RT-qPCR from independent biological replicates. These 12 selected miRNAs included highly expressed miRNAs, which were upregulated miRNAs (hsa-miR-192, hsa-miR-375, hsa-miR-451, hsa-miR-320a, hsa-miR-92a, hsa-let-7b and hsa-miR-181a) and downregulated (hsa-miR-21 and hsa-miR-221) and miRNAs with low levels of expression (hsa-let-7e, hsa-miR-29b and hsa-miR-219-5p) which were all downregulated in the serum, compared with plasma. As shown in Fig. 4B, a significant correlation (Pearson's correlation = 0.974) was revealed between the SOLiD deep sequencing data and the RT-qPCR measurements, indicating the robustness of deep sequencing based expression analysis.

### Predicted targets of differentially expressed miRNAs

The most abundant maternal plasma miRNAs were selected to analyze the differential expression of miRNA between the serum and plasma samples. Those miRNAs with sequencing counts ≥20 and an absolute fold change >2 or <0.5 in the serum compared with the plasma, were regarded as differentially expressed miRNAs.

To examine the biological function of the differentially expressed miRNAs identified, computational analysis was performed using TargetScan for the identification of predicted messenger RNA targets for each miRNA. Table II lists the predicted targets, in which a total of 3,198 genes were potential targets of these miRNAs. To examine the target gene functions, these predicated miRNA targets were annotated with GO and KEGG schemes using DAVID. The predicted miRNA targets populated several major GO categories and, for certain miRNAs, the number of gene targets was significantly increased (P<0.001 using Benjamini's correction). The molecular function, biological process and cellular component GO classifications, were evaluated, however, only significant terms of biological process are listed in Table II. The majority of the significant GO terms were associated with the regulation of transcription. There were also a number of significantly increased GO categories, including cell division, cell cycle and mitosis.

Among the predicted targets, transmembrane (TM) protein was involved in seven significantly upregulated miRNAs. TM, is a receptor of thrombin and can change its conformation, preventing blood from clotting.

To analyze the role of miRNAs in the regulatory networks, putative miRNA targets were assigned to KEGG pathways, which revealed 11 significantly increased pathways (P<0.001, using Benjamini's correction). The majority of these miRNAs were target genes involved in cell meiosis, cell cycle, leukemia, cancer and infection (Table III).

The present study identified, using next generation sequencing technology and RT-qPCR, a small set of miRNAs, which were differentially expressed between the serum and plasma of pregnant females. The differential miRNA suggested that different results may be obtained when using either serum or plasma in associated nucleic acid investigations and clinical applications. A previous study confirmed the differences between plasma and serum. Rudstein-Svetlicky *et al* revealed the existence of differentially expressed proteins, and suggested that plasma may be more suitable for diagnosis and investigations (24). Omenn *et al* demonstrated that, compared with serum, plasma maintains increased fidelity without *in vitro* coagulation (25). However, serum and plasma were generally selected arbitrarily in the majority of the previous studies, and no obvious differences were observed in these associated experiments (26,27). These findings suggested the importance of care in the selection of serum or plasma for future investigation and clinical use.

In the present study, peripheral blood samples from pregnant females were collected and sequenced. Pregnancy is a highly regulated complex physiological process, and the maternal body undergoes substantial changes to maintain a normal pregnancy (28). A previous study demonstrated that circulating miRNAs can be detected in samples from the serum and plasma. The stability of miRNAs is high, making the circulating miRNAs potentially useful candidates for diagnostic and other clinical applications (29). For these reasons, it is important to determine the differences in the expression levels of miRNAs between serum and plasma, and investigation of whether the miRNA expression pattern differs between the serum and plasma of patients and healthy individuals is required in the future.

The advent of high-throughput sequencing technology has reduced sequencing costs by orders of magnitude and has significantly increased the throughput. Whole-genome sequencing has become a technique for obtaining global genomic information about patients (30). In the present study, high-throughput sequencing was used to examine the entire expression pattern and differences in the expression of miRNA in maternal serum and plasma, however, to understand the difference in-depth, the functions of these differentially expressed miRNAs requires further analysis and confirmation.

The miRNA functions were analyzed using bioinformatic technologies. The experimentally validated target sites were collected from the miRTar-Base database (31) and those genes targeted by >2 miRNAs were regarded as target genes of maternal plasma miRNAs. The functions and functional associations of these target genes were analyzed using the KEGG database (32), and the results demonstrated that the increased pathways of the differentially expressed miRNA targets included the cell meiosis, cancer and cell cycle pathways. These pathways were closely associated with the progression of pregnancy, and a number were associated with blood coagulation. These results suggested that a number of the differentially expressed miRNAs in the serum, compared with the plasma, were involved in the process of blood coagulation and associated changes. The details of the underlying regulatory mechanism remain to be elucidated.

In conclusion, the present study sequenced and analyzed circulating miRNAs in the plasma and serum of pregnant females. Differentially expressed miRNAs were identified between these two libraries and a number of these miRNAs were involved in the process of blood coagulation. These results demonstrated that plasma may more suitable for further investigations into miRNA during pregnancy.

## Figures and Tables

**Table I:** miRNAs with the highest levels of expression (top 20) and miRNAs up/downregulated in the serum, compared with the plasma of pregnant females.

Upregulated in serum compared with plasma	Downregulated in serum compared with plasma	Most highly expressed miRNAs in plasma	Most highly expressed miRNAs in serum
hsa-miR-1308	hsa-miR-21	hsa-miR-221	hsa-miR-192
hsa-miR-192	hsa-miR-222	hsa-miR-222	hsa-miR-23a
hsa-miR-375	hsa-let-7e	hsa-miR-23a	hsa-miR-1308
hsa-miR-143	hsa-miR-221	hsa-miR-29a	hsa-let-7b
hsa-miR-451	hsa-miR-219-5p	hsa-miR-21	hsa-miR-29a
hsa-miR-145	hsa-miR-29b	hsa-miR-24	hsa-miR-24
hsa-miR-27b		hsa-miR-124	hsa-miR-22
hsa-miR-17		hsa-miR-22	hsa-miR-221
hsa-miR-199a-3p		hsa-let-7b	hsa-miR-451
hsa-miR-320a		hsa-miR-26a	hsa-miR-375
hsa-miR-92a		hsa-miR-192	hsa-miR-26a
hsa-miR-423-5p		hsa-let-7a	hsa-miR-145
hsa-miR-25		hsa-let-7f	hsa-let-7a
hsa-miR-29c		hsa-miR-23b	hsa-miR-92a
hsa-let-7b		hsa-miR-214	hsa-miR-143
hsa-miR-181a		hsa-miR-1308	hsa-miR-23b
hsa-miR-22		hsa-miR-181a	hsa-miR-222
hsa-let-7c		hsa-miR-451	hsa-miR-27b
hsa-miR-23a		hsa-miR-92a	hsa-miR-124
		hsa-miR-31	hsa-miR-320a

miR/miRNA, microRNA.

**Table II:** GO term predicted targets of differentially expressed microRNAs.

GO ID	GO Term	GO type	Hypergeometric P-value	Corrected P-value (BF)	Hit gene number for term
GO:0006355	Regulation of transcription, DNA-dependent	Process	5.664037e-10	1.137339e-06	159
GO:0051301	Cell division	Process	3.218192e-09	6.462130e-06	44
GO:0006366	Transcription from RNA polymerase II promoter	Process	8.628167e-08	1.732536e-04	43
GO:0006417	Regulation of translation	Process	1.726526e-07	3.466864e-04	17
GO:0016568	Chromatin modification	Process	1.988089e-07	3.992083e-04	36
GO:0007049	Cell cycle	Process	4.647136e-07	9.331448e-04	53
GO:0000278	Mitotic cell cycle	Process	7.049451e-07	1.415530e-03	43
GO:0006468	Protein phosphorylation	Process	1.558293e-06	3.129053e-03	48
GO:0006351	Transcription, DNA-dependent	Process	1.568039e-06	3.148622e-03	64
GO:0045893	Positive regulation of transcription, DNA-dependent	Process	2.264364e-06	4.546843e-03	50
GO:0044419	Interspecies interaction between organisms	Process	2.696993e-06	5.415562e-03	42
GO:0007067	Mitosis	Process	3.184293e-06	6.394060e-03	29

GO, gene ontology; BF, Bayesian factors.

**Table III:** KEGG term of the predicted targets of the differentially expressed microRNAs.

KEGG term	Hypergeometric P-value	Corrected P-value (BF)	Hit gene number for term
Pathways in cancer	4.165742e-08	7.789938e-06	47
Chronic myeloid leukemia	5.045730e-08	9.435516e-06	19
Prostate cancer	1.864796e-07	3.487168e-05	20
Cell cycle	4.543671e-07	8.496665e-05	24
Oocyte meiosis	6.022683e-07	1.126242e-04	22
Pancreatic cancer	9.149623e-07	1.710979e-04	17
Small cell lung cancer	2.688319e-06	5.027156e-04	18
Renal cell carcinoma	5.485690e-06	1.025824e-03	16
Non-small cell lung cancer	1.833852e-05	3.429304e-03	13
Glioma	2.430872e-05	4.545730e-03	14
HTLV-I infection	2.436786e-05	4.556789e-03	34

KEGG, Kyoto Encyclopedia of Genes and Genomes; BF, Bayesian factors.
